# Cobra Venom Factor Boosts Arteriogenesis in Mice

**DOI:** 10.3390/ijms23158454

**Published:** 2022-07-30

**Authors:** Philipp Götz, Sharon O. Azubuike-Osu, Anna Braumandl, Christoph Arnholdt, Matthias Kübler, Lisa Richter, Manuel Lasch, Lisa Bobrowski, Klaus T. Preissner, Elisabeth Deindl

**Affiliations:** 1 Walter-Brendel-Centre of Experimental Medicine, University Hospital, Ludwig-Maximilians-Universität München, 81377 Munich, Germany; p.goetz@med.uni-muenchen.de (P.G.); or sharon.eboagwu@funai.edu.ng (S.O.A.-O.); anna.braumandl@med.uni-muenchen.de (A.B.); christoph.arnholdt@med.uni-muenchen.de (C.A.); matthias.kuebler@med.uni-muenchen.de (M.K.); manuel_lasch@gmx.de (M.L.); lisa.bobrowski@med.uni-muenchen.de (L.B.); 2 Biomedical Center, Institute of Cardiovascular Physiology and Pathophysiology, Ludwig-Maximilians-Universität München, 82152 Planegg-Martinsried, Germany; 3 Department of Physiology, Faculty of Basic Medical Sciences, College of Medicine, Alex Ekwueme Federal University Ndufu Alike, Abakaliki 482131, Ebonyi, Nigeria; 4 Flow Cytometry Core Facility, Biomedical Center, Ludwig-Maximilians-Universität München, 82152 Planegg-Martinsried, Germany; l.richter@med.uni-muenchen.de; 5 Department of Otorhinolaryngology, Head and Neck Surgery, University Hospital, Ludwig-Maximilians-Universität München, 81377 Munich, Germany; 6 Department of Cardiology, Kerckhoff-Heart Research Institute, Faculty of Medicine, Justus Liebig University, 35392 Giessen, Germany; klaus.t.preissner@biochemie.med.uni-giessen.de

**Keywords:** arteriogenesis, complement system, C3, cobra venom factor, mast cells, macrophages, complement activation, neutrophils, platelets, platelets-neutrophil aggregates

## Abstract

Arteriogenesis, the growth of natural bypass blood vessels, can compensate for the loss of arteries caused by vascular occlusive diseases. Accordingly, it is a major goal to identify the drugs promoting this innate immune system-driven process in patients aiming to save their tissues and life. Here, we studied the impact of the Cobra venom factor (CVF), which is a C3-like complement-activating protein that induces depletion of the complement in the circulation in a murine hind limb model of arteriogenesis. Arteriogenesis was induced in C57BL/6J mice by femoral artery ligation (FAL). The administration of a single dose of CVF (12.5 µg) 24 h prior to FAL significantly enhanced the perfusion recovery 7 days after FAL, as shown by Laser Doppler imaging. Immunofluorescence analyses demonstrated an elevated number of proliferating (BrdU^+^) vascular cells, along with an increased luminal diameter of the grown collateral vessels. Flow cytometric analyses of the blood samples isolated 3 h after FAL revealed an elevated number of neutrophils and platelet-neutrophil aggregates. Giemsa stains displayed augmented mast cell recruitment and activation in the perivascular space of the growing collaterals 8 h after FAL. Seven days after FAL, we found more CD68^+^/MRC-1^+^ M2-like polarized pro-arteriogenic macrophages around growing collaterals. These data indicate that a single dose of CVF boosts arteriogenesis by catalyzing the innate immune reactions, relevant for collateral vessel growth.

## 1. Introduction

The vascular system has the fundamental role of delivering blood with its nutrients and oxygen to peripheral tissues. The devastating consequences of vascular occlusive diseases such as myocardial infarction, peripheral artery disease, stroke, or even loss of an artery due to abdominal aortic aneurism (AAA) surgery might be prevented by the timely induction of collateral artery growth to form natural bypasses [1,2,3,4]. This highly complex and multifactorial process, which is particularly driven by changes in fluid shear stress of affected vessels, is known as arteriogenesis [5,6]. This multistep process is described as the remodeling of pre-existing arterio-arteriolar anastomoses into completely developed functional arteries, characterized by the proliferation of endothelial and smooth muscle cells and promoted by the cellular and humoral components of the innate immune system [7,8].

In detail, it has been shown that, upon occlusion of a supplying artery, the blood flow is redirected into pre-existing arteriolar connections where the arising increased fluid shear stress elicits a local and timely well-coordinated sterile inflammatory process. Upon this mechanical stress, endothelial cells release the von Willebrand factor (vWF), which promotes the activation of platelets. Subsequently, these activated platelets interact with neutrophils to form platelet-neutrophil aggregates (PNA) with the concomitant activation of NADPH oxidase 2 (Nox-2) in neutrophils. Upon adhesion on intercellular adhesion molecule-1 (ICAM-1) and urokinase plasminogen activator (uPA)-mediated extravasation of neutrophils—both proteins are increased, expressed in activated endothelial cells of growing collaterals [9,10]—neutrophil-derived reactive oxygen species (ROS) activate perivascular mast cells. These cells, in turn, create an inflammatory microenvironment, resulting in the further recruitment of neutrophils, as well as macrophages, the latter supplying growth factors and cytokines to the growing collateral vessel (for a review, see [11]). Interestingly, it has been demonstrated that especially CD68+/MRC-1+ M2-like polarized macrophages, which play a major role in tissue remodeling and the resolution of inflammation, are of major relevance for an effective arteriogenesis [12,13].

The complement system is an important part of the innate immune system playing a role in the resistance against infection and clearance of altered host cells. Complement components also have regulating properties in inflammatory and immune responses and thereby are key players in the pathogenesis of several diseases, like shock, stroke, immune complex diseases, autoimmune hemolytic anemia, or asthma [14]. The complement system is composed of about 30 plasma and cell surface proteins, numerous receptors, and regulatory factors expressed in the membranes of various cell types, such as platelets, endothelial cells, and cells of the immune system [15,16]. These proteins allow to recognize pathogens and eradicate them from the host’s system [17].

The complement system can be canonically activated via three specific, well-defined pathways: the classical, the lectin and the alternative pathway. These three pathways share a similar molecular architecture, including somewhat different primary recognition and activation events that are magnified by proteolytic reactions converging at the point of C3 cleavage/activation [18]. As an effective chemoattractant, the resulting small C3a peptide serves to concentrate neutrophils at the inflamed site, whereas the large C3b fragment is incorporated into the proteolytic convertases to amplify the complement activation. Finally, in the terminal phase, the membrane attack complex (MAC) is assembled on the surface of pathogens to obtain cytolytic poly-C9 pores as an effective killing mechanism [18]. However, it should be mentioned that the complement system can be activated noncanonically [19].

Cobra venom factor (CVF) is a complement-activating protein, which has been isolated from venom of the cobra [15]. It is functionally homologous to C3b, the activated form of C3, and accordingly binds factor B, to be subsequently cleaved by factor D to form the bimolecular complex CVF–Bb, which is a C3/C5 convertase. CVF–Bb cleaves both C3 and C5 but is resistant to inactivation by the regulating factors of the complement system such as Factor H and due to its special structure, more stable than its physiological pendant [20]. As CVF can cleave C3 almost completely, it has been used for many years to deplete the serum complement (C3) in animals in a bid to delineate the biological functions of the complement system and its role in the pathogenesis of many diseases [18]. CVF is used as an important tool to target complement activation, and no adverse effects were observed in animals, except neutrophil sequestration to the lungs [21]. CVF is not a toxin, but it can activate the alternative pathway. However, it only consumes complement (C3) and effectively depletes the complement activity in the serum of treated animals [15]. A great deal of effort has been invested in the development of drugs for complement inhibition [22]. These drugs inhibit complement activation, whereas CVF depletes complement C3.

Despite the fact that humoral and cellular components of the innate immune system play a major role in arteriogenesis, the contribution of the complement remains obscure. Since the generation of collateral vessels is required in situations of artery occlusion, one is left to promote collateral artery growth in clinical practice. This study will provide information about the application and usefulness of CVF for the process of arteriogenesis as an option for therapeutical intervention in patients with vascular occlusive diseases. We investigated the possible mechanisms by which CVF through complement activation and consumption may have an impact on arteriogenesis. The obtained results indicate that treatment with a single dose of CVF 24 h prior to artery occlusion results in an enhanced growth and formation of collateral vessels in mice. While these data provide further insight into the contribution of innate immunity for the process of arteriogenesis, it remains to be investigated whether CVF administration could be available as a medical treatment.

## 2. Results

### 2.1. Treatment with CVF in the Experimental Mouse Model of Arteriogenesis

**Figure 1:**
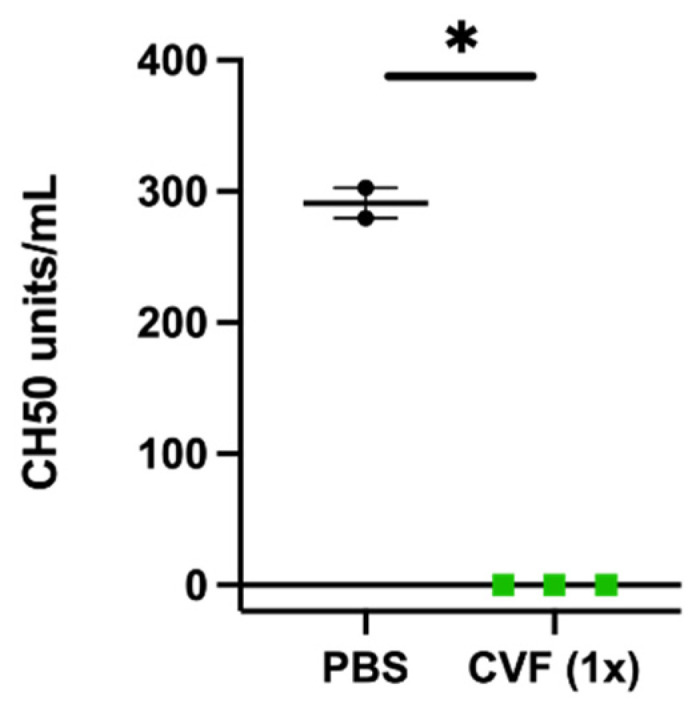
Single-dose application of 12.5 µg of the cobra venom factor (CVF (1x)) leads to the loss of hemolytic complement activity of the serum 24 h after injection. The scatter plot represents the results of a CH50 assay. Data are the means ± S.E.M., *n* = 3 for the CVF-treated group, *n* = 2 for the PBS-treated control group, * *p* < 0.05 (PBS vs. CVF (1x)) by an unpaired Student’s *t*-test.

To study the influence of the complement system and the impact of CVF on arteriogenesis, we used a well-established murine hind limb model to induce collateral vessel formation [8]. In this model, the unilateral ligation of the right femoral artery results in the growth of collateral blood vessels (arteriogenesis) in the adductor muscle of the upper leg. As an internal control, the left femoral artery was sham-operated. To ascertain the efficacy of CVF in our animal model, 24 h prior to femoral artery ligation (FAL), C57Bl/6J mice underwent i.p. injection of either a single dose of 12.5 µg of CVF dissolved in 50 µL PBS or an equivalent volume of PBS (control group). The complement consumption impact of the treatment was proven by measuring the 50% hemolytic complement activity of the serum before starting the FAL (Figure 1).

#### 2.1.1. Influence of CVF on Perfusion Recovery

**Figure 2:**
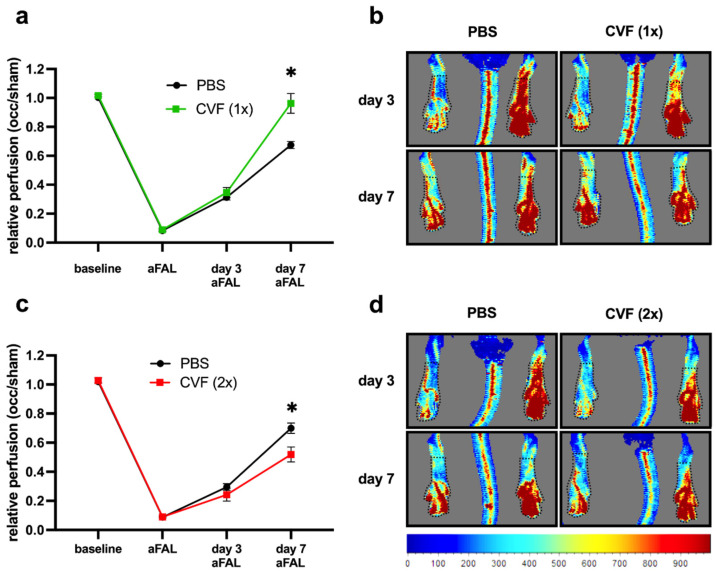
Impact of CVF administration on the perfusion after femoral artery ligation (FAL). (**a**,**b**) Single-dose application of the cobra venom factor (CVF (1x)) promotes perfusion recovery. (**a**) The line graph describes laser Doppler perfusion measurements of the hindlimbs from mice treated with a single dose of phosphate-buffered saline (PBS) or CVF (1x) 24 h prior to FAL. The relative perfusion was calculated using an occluded/sham (right to left hind limb) ratio before FAL (baseline), directly after FAL (aFAL), at day 3, and at day 7 aFAL. Data are the means ± S.E.M., *n* = 5 mice per group, * *p* < 0.05 (PBS vs. CVF (1x)) by two-way analysis of variance (ANOVA) with the Bonferroni multiple comparison test. (**b**) Representative flux images of laser Doppler measurements from mice treated with a single dose of PBS (left images) or CVF (1x) (right images) showing color-coded the relative perfusion of hind limbs at day 3 and 7 aFAL (blue color indicates low perfusion, and red color indicates high perfusion). Black dotted lines indicate the regions of interest (ROI), which were used for perfusion analyses. (**c**,**d**) Repeated treatment of mice with CVF (2x) counteracts the perfusion recovery. (**c**) The line graph depicts the results of the laser Doppler perfusion measurements of the hind limbs of repeated PBS- or CVF (2x)-treated mice. Some (12.5 µg CVF) were administered by i.p. injection 24 h prior FAL and again 3 days aFAL. The relative perfusion was calculated using the occluded/sham (right to left hind limb) ratio before FAL (baseline), directly aFAL, at day 3, and at day 7 aFAL. Data are the means ± S.E.M., *n* = 5 per group, * *p* < 0.05 (PBS vs. CVF (2x)) by two-way analysis of variance (ANOVA) with the Bonferroni multiple comparison test. (**d**) Representative flux images of laser Doppler measurements from repeated PBS- (left images) or CVF (2x)-treated mice (right images) showing in color code the relative hindlimb perfusion at day 3 and 7 aFAL. Black dotted lines indicate the ROI, which were used for perfusion analyses.

To measure the influence of CVF on the perfusion recovery after FAL, mice underwent laser Doppler perfusion measurements of their hindlimbs before, directly after, 3 days, and 7 days after ligation. In mice receiving a single dose of CVF 24 h before the induction of arteriogenesis via FAL, we observed a significantly improved perfusion recovery compared to the PBS-treated control group 7 days after the surgical procedure (Figure 2a,b). However, when the mice were treated twice, i.e., 24 h before and again 3 days after FAL, with CVF, a negative impact on the perfusion recovery was seen (Figure 2c,d).

#### 2.1.2. Influence of Treatment with a Single Dose of CVF on Luminal Diameter and Vascular Cell Proliferation

**Figure 3:**
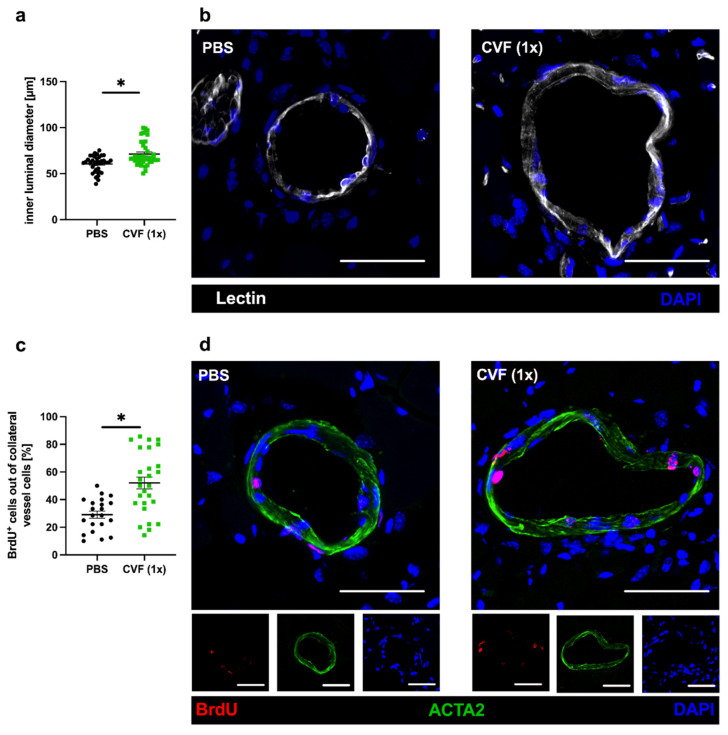
Single-dose application of the cobra venom factor (CVF (1x)) 24 h prior to femoral artery ligation (FAL) enhances collateral growth. The scatter plots describe (**a**) the inner luminal diameter of proliferating collaterals and (**c**) the number of proliferating vascular cells (BrdU+, bromodeoxyuridine+ cells) per growing collateral in percent of phosphate-buffered saline (PBS)- and CVF-treated mice 7 days after FAL. Data are the means ± S.E.M., *n* = 5 mice per group, *n* > 10 values per group, * *p* < 0.05 (PBS vs. CVF (1x)) by the unpaired Student’s *t*-test. (**b**,**d**) Representative immunofluorescence pictures of growing collaterals collected from PBS-treated (left panels) and CVF (1x)-treated (right panels) mice 7 days after ligation stained with lectin (white), indicating the luminal vessel boundary (**b**, **upper** images), as well as antibodies against BrdU (red), marking proliferating cells and ACTA2 (green) staining smooth muscle cells (**d**, **lower** images). DAPI (blue) was used to label the nuclei; scale bar: 50 µm.

To confirm that the improved perfusion recovery observed after administration of a single dose of CVF was due to enhanced collateral artery growth (and not due to simple vasodilation), we performed immunofluorescence analyses of the adductor muscle by studying the inner luminal diameter and the number of proliferating vascular cells 7 days after FAL. Lectin was implemented to mark the inner vascular cell layer for the analysis of the luminal diameter. Bromodeoxyuridine (BrdU) served as a proliferation marker, while ACTA2 as smooth muscle cell marker was used to mark the outer vascular boundary for the proliferation analysis. Thus, BrdU^+^ cells within the vascular structure were counted as proliferating vascular cells, whereby BrdU signals in ACTA2^+^ cells were counted as proliferating vascular smooth muscle cells, and BrdU signals at luminal ACTA2^−^ structures as proliferating endothelial cells. However, for quantification, we did not distinguish between these subpopulations of vascular cells. CVF (1x)-treated mice showed a significantly increased inner luminal diameter of the growing collaterals compared to the PBS-treated control group (Figure 3a,b). Additionally, there was a significant increase in the number of proliferating collateral vascular cells in the CVF (1x)-treated group compared to the PBS-treated controls (Figure 3b,d). Investigations on sham-operated legs showed no differences either in the luminal diameter or proliferating vascular cells between CVF- and PBS-treated mice (data not shown).

#### 2.1.3. Single-Dose Application of CVF Enhances Neutrophil Mobilization and PNA Formation

**Figure 4:**
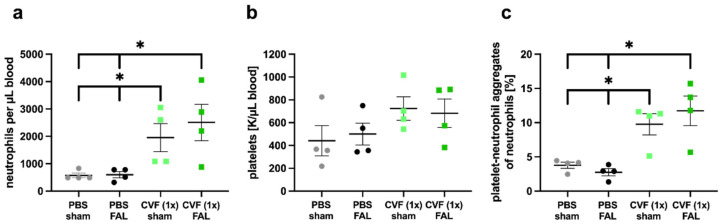
Single-dose application of the cobra venom factor (CVF (1x)) enhances the formation of platelet-neutrophil aggregates (PNAs) and the mobilization of neutrophils to the peripheral blood. Scatter plots show flow cytometry analyses of (**a**) the number of neutrophils per microliter of blood, (**b**) the number of platelets per microliter of blood measured by the differential blood count, and (**c**) the percentage of PNA formation relative to the total number of neutrophils in phosphate-buffered saline (PBS)- and CVF (1x)-treated mice 27 h after injection, respectively, 3 h after double femoral artery ligation (FAL) or a double sham operation (sham) of both femoral arteries. For the PNA analysis, platelets were detected by an anti-CD41 antibody, and neutrophils were identified by anti-CD11b and anti-Gr-1 antibodies. All values are the means ± S.E.M., *n* = 4 mice per group, * *p* < 0.05 by one-way analysis of variance (ANOVA) with Tukey’s multiple comparison test.

As PNA formation is an important prerequisite for effective arteriogenesis, we investigated the number of platelets and neutrophils, as well as their complexes (expressed as PNAs), as early as 3 h after FAL and 27 h after treatment with a single dose of CVF or PBS, respectively. The influence of CVF on neutrophil and platelet counts, as well as PNA formation, was analyzed in blood samples of PBS- and CVF (1x)-treated mice that either underwent ligation of the right and left femoral arteries or received a sham operation of both legs. Interestingly, we found a significantly increased number of neutrophils and PNAs in mice treated with a single dose of CVF compared to the PBS-treated control groups, irrespective of whether the mice experienced femoral artery ligation or a sham operation (Figure 4a,c). The platelet count was slightly increased in CVF (1x)-treated mice; however, it did not show significantly different values (Figure 4b).

#### 2.1.4. Single-Dose CVF Treatment Results in Increased Perivascular Mast Cell Recruitment and Activation

**Figure 5:**
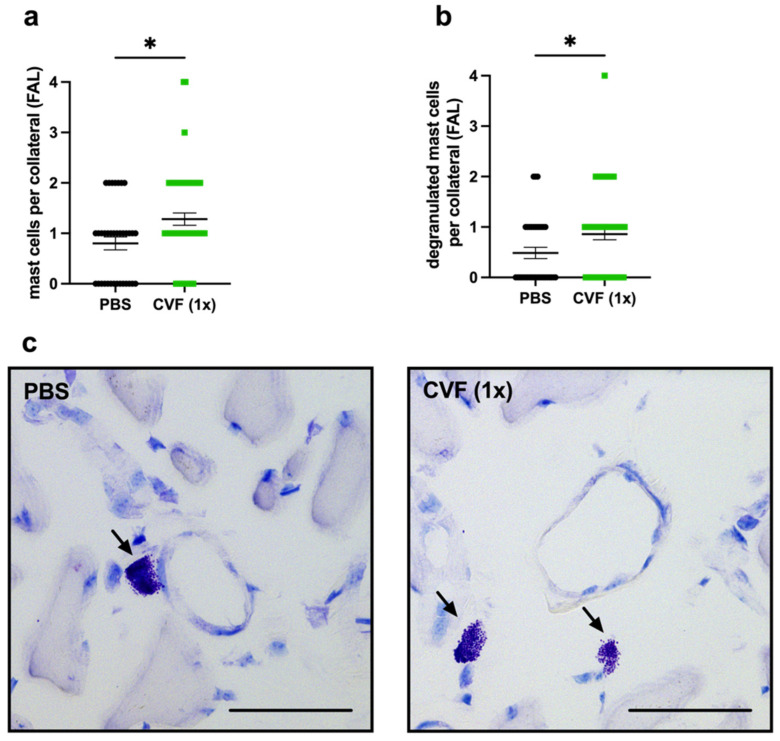
Single-dose application of the cobra venom factor (CVF 1x)) increases the number of perivascular mast cells and promotes the degranulation of mast cells around growing collateral arteries. The scatter plots represent (**a**) the number of perivascular mast cells per collateral in the adductor muscles 8 h after femoral artery ligation (FAL) of phosphate-buffered saline (PBS)- and CVF-treated mice, as well as (**b**) the number of degranulated mast cells per collateral 8 h after FAL. Data are the means ± S.E.M., *n* = 3 mice per group, *n* > 10 values per group, * *p* < 0.05 (PBS vs. CVF (1x)) by the unpaired Student’s *t*-test. (**c**) Representative Giemsa stains depicting mast cells (arrows) in the perivascular space of collateral vessels of PBS- (left image) and CVF (1x)-treated (right image) mice 8 h after FAL. Scale bar: 50 µm.

As already shown in a previous study, mast cell activation is essential for arteriogenesis [8]. Here, we assessed the total number, as well as the number of degranulating mast cells, in the perivascular space of the proliferating and resting collaterals by Giemsa staining of the adductor muscle tissue collected 8 h after the FAL or sham operation. Comparing the adductor muscles from the femoral artery ligated site of single-dose CVF- with PBS-treated mice, we found significantly more mast cells in the perivascular space of the collaterals collected from CVF (1x)-treated mice (Figure 5a,c). Furthermore, we observed an increase of degranulated mast cells per collateral in mice that underwent the single-dose CVF treatment (Figure 5b,c). However, no significant difference concerning the number of mast cells was seen in the perivascular space of single-dose CVF vs. PBS-treated sham-operated mice, although, here, the number of degranulated mast cells was increased as well (Supplementary Materials Figure S1).

#### 2.1.5. Single-Dose CVF Treatment Promotes Regenerative M2-like Macrophage Polarisation

**Figure 6:**
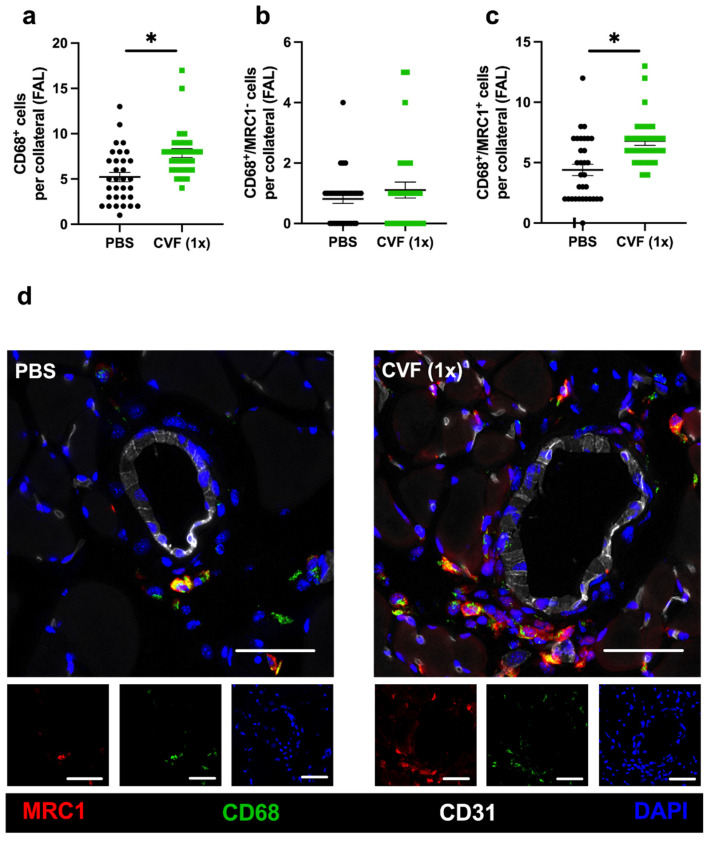
Single-dose cobra venom factor (CVF (1x)) application leads to an increased number of M2-like polarized macrophages per growing collateral. The scatter plots display (**a**) the number of perivascular CD68^+^ cells (macrophages), (**b**) the number of CD68^+^/MRC1^−^ (mannose receptor C-type 1) cells per growing collateral, and (**c**) the number of CD68^+^/MRC1^+^ cells per growing collateral in the adductor muscle of single-dose phosphate-buffered saline (PBS)- or CVF-treated mice collected 7 days after femoral artery ligation (FAL). Data are the means ± S.E.M., *n* = 5 mice per group, *n* > 10 values per group, * *p* < 0.05 (PBS vs. CVF (1x)) by the unpaired Student’s *t*-test. (**d**) Representative immunofluorescence images of adductor muscles of single-dose PBS- (left images) or CVF-treated (right images) mice collected 7 days after FAL are presented. Images of single and merged channels show macrophages labeled with antibodies against CD68 (green) and MRC1 (red) in the perivascular space of the collaterals. The endothelial cell marker CD31 (white) was used to depict collaterals and DAPI (blue) to label nucleic DNA; scale bar: 50 µm.

Macrophages and their polarization state play a pivotal role in arteriogenesis. To analyze macrophages in the perivascular space of the growing and resting collaterals, we used an anti-CD68 antibody to stain macrophages, together with an anti-mannose receptor c-type 1 (MRC1) antibody as a marker for the anti-inflammatory and regenerative M2-like polarization phenotype. Hence, CD68^+^/MRC1^−^ cells were quantified as proinflammatory M1-like polarized macrophages, whereas CD68^+^/MRC1^+^ cells were counted as anti-inflammatory M2-like polarized macrophages. We found a significantly increased number of perivascular macrophages per growing collateral in the adductor muscles of mice treated with a single dose of CVF compared to control mice 7 days after femoral artery ligation (Figure 6a). Hereby, the number of M1-like polarized macrophages (CD68^+^/MRC1^−^) per collateral did not differ between both experimental groups, whereas a significant increase in the number of M2-like polarized perivascular macrophages (CD68^+^/MRC1^+^) per collateral in CVF (1x)-treated mice compared to PBS-treated mice was observed (Figure 6b,c). No differences regarding the number of perivascular macrophages per resting collateral or their polarization state was noted in the sham-operated adductor muscles from both experimental groups (Supplementary Materials Figure S2).

## 3. Discussion

Arteriogenesis is a shear stress driven process that is supported by the immune system. Using a murine hind limb model, we show that a single dose of CVF boosts arteriogenesis by activating innate immune reactions. We demonstrate that a single-dose treatment with CVF results in the mobilization of neutrophils to the peripheral blood and an increased PNA formation, as well as an enhanced recruitment and activation of the mast cells, all of which are prerequisites for effective arteriogenesis. Moreover, we report a raised number of pro-arteriogenic M2-like polarized macrophages in the perivascular space of growing collateral arteries. Consequently, mice treated with a single dose of CVF displayed increased vascular cell proliferation, enlarged collateral diameters, and increased perfusion recovery upon femoral artery occlusion, making CVF a potent drug to foster collateral artery growth.

The influence of the complement system and different complement factors has been extensively studied in the context of angiogenesis [23,24,25]. In addition, CVF as a complement consuming drug has been investigated in various settings of inflammation in animal experiments [18,26]. However, the role of the complement system in arteriogenesis has only started to be looked at [27], and CVF treatment has never been a topic in settings of experimental collateral growth.

There is plethora of data available in the literature suggesting that the complement system is involved in the process of arteriogenesis. For example, it has been described that endothelial cells are an extrahepatic source of components of the complement system, such as properdin, and that shear stress can cause their increased expression and release [28,29,30]. Properdin positively regulates the alternative pathway of the complement system by stabilizing C3 and C5 convertases. C3b can bind to ultra-large vWF—a protein that is released due to shear stress in growing collaterals [7]—and thereby locally activates the alternative pathway to generate a large amount of effector proteins [31,32]. Moreover, high concentrations of C3a and C5a can stimulate endothelial cells locally to express adhesion molecules for leukocytes such as intercellular adhesion molecule-1 (ICAM-1) [33] and leukocyte-recruiting chemokines such as monocyte chemoattractant protein-1 (MCP-1) [34]. Indeed, we have previously shown that an increased expression of ICAM-1 occurs in growing collaterals, contributing to arteriogenesis [9,35]. Moreover, we have shown that arteriogenesis is associated with increased levels of MCP-1 and that the administration of MCP-1 strongly promotes arteriogenesis [35].

To investigate the impact of CVF on arteriogenesis, we used a murine hind limb model of collateral artery growth to administer CVF either once or twice prior to FAL. When mice were treated twice with CVF, 24 h before induction of arteriogenesis by FAL and again 3 days after FAL, reduced arteriogenesis was noted, as evidenced by the LDI measurements. However, when mice were treated only once with CVF, namely 24 h before the induction of collateral artery growth, arteriogenesis was significantly improved, as confirmed by an increased luminal diameter and cell proliferation in growing collateral arteries 7 days after FAL.

The administration of CVF results within hours in decomplementation and has been demonstrated to reach its maximum after 24 h in mice [36]. A feasible test to investigate the effectiveness of CVF is a hemolytic assay [37,38]. Upon activation of the complement system, the membrane attack complex (MAC) is formed, which lyses erythrocytes. Accordingly, decomplementation results in a severe reduction in hemolytic activity. The decomplementating effect of CVF compared to PBS was proven in our study in the serum of mice 24 h after administration of a single dose of CVF.

In terms of the mobilization of leukocytes in New Zealand white rabbits, CVF administration showed a biphasic and opposing effect, since the application of CVF resulted in leukopenia within seconds, followed by leukocytosis with an almost fivefold increase in cell number (mainly of neutrophils) within 24 h [39]. Additional studies evidenced that the increased neutrophil count is due to the chemotactic recruitment of cells from the bone marrow as a consequence of increased C5a upon CVF treatment [40,41,42]. We here report a significant increase in the number of neutrophils 27 h after administration of a single dose CVF in mice, which was associated with an increased platelet–neutrophil aggregate formation. Interestingly, at that time point, which corresponds to 3 h after the onset of the surgical procedure of FAL, we found no changes in the number of PNA formation compared to the sham-operated PBS-treated mice. However, our results evidenced significant rises in PNA formation in both FAL and sham-operated mice after treatment with a single dose of CVF compared to the PBS treatment.

Increased PNA formation is a prerequisite for effective arteriogenesis, as it results in neutrophil, as well as subsequent mast cell, activation [7,8]. Indeed, CVF treatment resulted in an increased recruitment of mast cells in the perivascular space of growing collaterals already 8 h after FAL, which was not seen in sham-operated animals. In a previous study, we demonstrated that an increasing mast cell recruitment by treating mice with diprotin A (a dipeptidyl-peptidase IV (DPPIV) inhibitor that retards stroma cell-derived factor-1α, SDF-1α, degradation) resulted in improved arteriogenesis by recruiting SDF-1 receptor CXCR-4-expressing mast cells to the growing collaterals [8]. Additionally, PNA formation is a marker for platelet activation [43], and platelets are a rich source of SDF-1α [44,45], indicating that, upon CVF treatment, increased mast cell recruitment may be due to an enhanced PNA formation. Yet, as C3a and C5a are also chemo-attractants for mast cells [46], the CVF treatment could have contributed to the emergence of these anaphylatoxins to contribute to mast cell recruitment as well.

Under (patho-)physiological conditions, mast cell activation in the perivascular space of growing collaterals is largely mediated by neutrophil-derived ROS [8]. This is also very likely the case in CVF-treated mice, as these mice showed up with more PNA formation, which is associated with increased Nox-2 activation and, hence, ROS formation in neutrophils [8]. Moreover, anaphylatoxins produced by CVF application are able to trigger an oxidative burst of neutrophils [47]. Additionally, by binding to their respective receptors, C3a and C5a can directly promote mast cell activation [48,49]. We indeed found an increased number of activated mast cells in the perivascular space of sham-operated mice; however, mast cell activation did not result in vascular cell proliferation and subsequent collateral artery growth. The initial trigger for arteriogenesis is increased fluid shear stress, which is elicited in preexisting collateral arteries by redirected blood flow and results in endothelial cell activation [1]. Inter alia, this results in the increased expression of uPA in the wall of preexisting collaterals, which is essential not only for the extravasation of neutrophils to the perivascular space [50] but also important for monocyte transmigration, which, once matured to macrophages, are crucial for effective arteriogenesis [1,6]. Accordingly, CVF treatment did not result in an increased accumulation of CD68^+^ macrophages around collateral arteries of sham-operated legs, and the sham operation was not associated with increased vascular cell proliferation or the luminal diameter in preexisting collaterals, indicating that the CVF treatment itself is not a direct growth stimulus for collaterals under non-pathophysiological conditions.

The pivotal role of macrophages in arteriogenesis is well-known, as has been shown in several previous studies [51,52]. By providing growth factors and remodeling the perivascular space, they have a huge impact on vascular cell proliferation and artery growth [53,54]. In the current study, mice treated with a single dose of CVF showed a higher macrophage accumulation in the perivascular space of growing collaterals, which was based on a higher number of M2-like polarized macrophages.

## 4. Materials and Methods

### 4.1. Animals and Experimental Procedures

Permissions for in vivo experiments and animal use were obtained from the Bavarian Animal Care and Use Committee (ethical approval code: ROB-55.2Vet-2532.Vet_02-17-99), and the performances of all experiments were in strict accordance with German and NIH animal legislation guidelines. The experiments included the use of C57BL/6J mice provided by Charles River (Sulzfeld, Germany). Eight-to-twelve-week-old mice were anesthetized with a combination of fentanyl (0.05 mg/kg, CuraMED Pharma, Karlsruhe, Germany), midazolam (5.0 mg/kg, Ratiopharm GmbH, Ulm, Germany), and medetomidine (0.5 mg/kg, Pfister Pharma, Berlin, Germany). The right femoral artery was ligated to set the stimulus, leading to arteriogenesis in the adductor muscle, whereas the left leg was sham-operated, as previously described [55]. As the proliferation marker, bromodeoxyuridine (BrdU) was administered daily i.p. (1.25 mg per day, Sigma-Aldrich, St. Louis, MO, USA) dissolved in 100 µL phosphate-buffered saline (PBS, 148 mM Na^+^, 1.8 mM K^+^, pH 7.2) starting immediately after femoral artery ligation (FAL). One day prior to surgery, cobra venom factor (REF A600, Quidel Co., Athens, OH, USA) was administered as a single-dose intraperitoneal (i.p.) injection with 12.5 µg diluted in 50 µL PBS. A PBS i.p. dose of the same volume served as the control. The perfusion of the hind limb was measured by Laser Doppler Imaging (LDI) using a Moor LDI 5061 and Moor Software Version 3.01 (Moor Instruments, Remagen, Germany). Prior to measurements, the body temperature was controlled for 10 min and kept between 36 °C and 38 °C during the experiments. LDI was performed before ligation (baseline) and directly after FAL, as well as 3 and 7 days after FAL. A flux mean value of a defined region (0.45 cm^2^) starting from the ankle to the toes of each animal was calculated. The perfusion was calculated by flux means of the ligated (right)-to-sham (left)-operated ratios.

Tissues were collected for histological analyses at intervals of 8 h or 7 days after FAL. During surgery and the experimental period until the collection of the tissue, none of the mice died or showed signs of infection or necrosis. To study the formation of platelet neutrophil aggregates, blood was collected 3 h after ligation of both femoral arteries (right) and the sham operation (left). Mice were sacrificed using cardiac puncture under deep narcosis. The bodies were perfused with 1% adenosine buffer (Sigma-Aldrich, Taufkirchen, Germany) containing 5% bovine serum albumin (BSA, Sigma-Aldrich, Taufkirchen, Germany) and dissolved in PBS, followed by 3% paraformaldehyde (PFA, Merck, Darmstadt, Germany). Finally, the adductor muscles were collected, kept in a 30% sucrose solution overnight, and then stored in vinyl molds (REF 4566, Sakura Finetek, Torrance, CA, USA) on Tissue-Tek^®^ (REF 4583, Sakura Finetek, Torrance, CA, USA) at −80 °C until further use.

### 4.2. Histological and Immunofluorescence Analyses

For histology, staining was performed with 8-µm-thick cryosections of the adductor muscle. Tissues of day 7 after FAL were used to stain for vascular cell proliferation, inner luminal diameter analysis, and the presence of macrophages. For cell proliferation, the sections were incubated with 1N HCl for 30 min at 37 °C to bare BrdU in the nuclei, blocked with 10% goat serum, dissolved in 4% BSA PBS/0.1% Tween-20 (Tween 20, AppliChem GmbH, Darmstadt, Germany) for 1 h at room temperature (RT), and then incubated with an anti-BrdU antibody (1:50, Abcam, Cambridge, UK, ab6326) at 4 °C overnight. Goat anti-rat Alexa Fluor^®^ 546 antibody (1:100, Thermo Fischer, A-11081, Rockford, IL, USA) was used as a secondary antibody, and anti-ACTA2-Alexa Flour^®^ 488 (anti-actin alpha 2, 1:400, Sigma-Aldrich, F3777, Saint-Louis, MO, USA) was used to mark the outer vessel layer.

Macrophages in tissues were labeled with anti-CD68-Alexa Fluor® 488 (1:200, Abcam, ab201844) and anti-MRC1 antibody (1:200, Abcam, ab64693) at 4 °C overnight, followed by the secondary antibody donkey anti-rabbit-Alexa Fluor® 546 (1:200, Thermo Fisher, A10040). An Alexa Fluor® 647 anti-mouse CD31 antibody (1:100, BioLegend, San Diego, CA, USA, 102516) was used to label the vascular cells. Additionally, all tissues were incubated with DAPI (1:1000, Thermo Fisher, ord. no. 62248, Rockford, IL, USA) for 10 min at RT to label the nucleic DNA. Dako mounting medium (Dako, Agilent, Santa Clara, CA, USA) was used to mount the stained slides. Giemsa stain was performed following the standard protocols. The mounted tissues of CVF-treated mice and control mice were analyzed using an epifluorescence microscope DM6 B (Leica microsystems, Wetzlar, Germany) for dark and bright field imaging. The open-source program ImageJ was used for counting analyses and inner luminal diameter analyses. Several sections were analyzed per muscle.

To measure the luminal diameter of the collaterals, the sections were stained with lectin (5 µg/mL, Sigma-Aldrich, L4895, Saint-Louis, MO, USA) for 2 h at RT.

### 4.3. Blood Analyses

Blood was collected via cardiac puncture with a standardized amount of anticoagulation (10 UE heparin per mL blood). For the flow cytometry analysis, 100 µL of full blood was lysed in 2 mL lysing solution (1:10 in aqua, BD Biosciences, 349202), centrifuged, and resuspended in a staining solution containing FITC anti-mouse CD41 (1:400, BioLegend, 133903), PE anti-mouse CD11b (1:300, BioLegend, 101208), Brilliant Violet 421™ anti-mouse CD115 (1:300, BioLegend, 135513), APC anti-mouse Ly-6G/Ly-6C (1:800, BioLegend, 108412), and eBioscience™ Fixable Viability Dye eFluor™ 780 (1:1000, Invitrogen, 65-0865-14). The samples were incubated for 20 min at 4 °C, washed, and subsequently analyzed with a BD LSRFortessa™ cell analyzer. Gating and analysis were performed using FlowJo V10. A full blood count was done with a ProCyte Dx using mouse-specific settings.

For the CH50 assay, measuring the complement lysing capacity, blood was collected without anticoagulant 24 h after treatment with a single dose of CVF or PBS to achieve clotting and serum generation for 1 h at room temperature. The probes were centrifuged at 706× *g* for 10 min at 4 °C. The supernatant was collected and stored at −80 °C. The CH50 assay was performed using 100 million antibody-sensitized sheep erythrocytes (Complement Technology, Inc., Tyler, TX, USA, B200). Five microliters of a Mouse Complement Assay Reagent (MCAR, Complement Technology, Inc., Tyler, TX, USA, B250) was used to improve the mouse CH50 titers. The serum samples were diluted to a final range from 1/163 to 1/650 in a veronal buffer (GVB++, Complement Technology, Inc., Tyler, TX, USA, B102). Controls included two samples with no serum as the background control and two samples containing sheep erythrocytes diluted in deionized water in place of the buffer for the 100% lysis control. Samples were handled on wet ice, and lysis was performed at 37 °C for 30 min. The remaining cells were spun down at 500× *g* for 3 min, and the absorbance of the supernatant was determined at 541 nm in a 1-cm cuvette. CH50 values (CH50 units/mL) represent the reciprocal value of the amount of serum able to lyse 50% of the sheep erythrocytes. Samples without lysing activities were set at 0 CH50 units/mL.

### 4.4. Statistical Analyses

The results were analyzed with GraphPad Prism 8 (GraphPad Software, LA Jolla, CA, USA) using the unpaired Student’s *t*-test or two-way analysis of variance (ANOVA) with the Bonferroni multiple comparison test. All data were presented as the mean values ± standard error of the mean (SEM). The findings were considered statistically significant at *p* < 0.05.

## 5. Conclusions

In summary, our data indicate that the application of a single dose of CVF 24 h prior to induction of arteriogenesis by FAL significantly promotes collateral vessel growth, as shown by increased perfusion recovery based on enhanced vascular cell proliferation and, hence, an increased collateral diameter. The administration of CVF already prior to the induction of arteriogenesis resulted in a massive mobilization of neutrophils to the peripheral blood and an increased PNA formation already at the time point when arteriogenesis was induced by FAL. Accordingly, we conclude from study that the prerequisites for efficient arteriogenesis, which are especially important for mast cell recruitment and activation—which, in turn, are essential for monocyte recruitment during the process of arteriogenesis—were already present and available at the moment when collateral artery growth was elicited by increased fluid shear stress. Thus, in situations where arteriogenesis is artificially promoted, e.g., prior to abdominal aortic aneurism surgery [3], the timely administration of CVF may be of particular interest. This could become a particular pro-arteriogenic treatment in patients since a humanized form of CVF already exists and would be ready to use [15,56,57,58].

## Data Availability

The data presented in this study are available on request from P.G. and A.B.

## Supplementary Materials

The following supporting information can be downloaded at: https://www.mdpi.com/article/10.3390/ijms23158454/s1.

## References

[1] Deindl E., Schaper W. (2005). The art of arteriogenesis. Cell Biochem. Biophys..

[2] Petroff D., Czerny M., Kölbel T., Melissano G., Lonn L., Haunschild J., Von Aspern K., Neuhaus P., Pelz J., Epstein D.M. (2019). Paraplegia prevention in aortic aneurysm repair by thoracoabdominal staging with ‘minimally invasive staged segmental artery coil embolisation’ (MIS²ACE): Trial protocol for a randomised controlled multicentre trial. BMJ Open.

[3] Etz C.D., Kari F.A., Mueller C.S., Brenner R.M., Lin H.-M., Griepp R.B. (2011). The collateral network concept: Remodeling of the arterial collateral network after experimental segmental artery sacrifice. J. Thorac. Cardiovasc. Surg..

[4] Stoller M., Seiler C. (2017). Effect of Permanent Right Internal Mammary Artery Closure on Coronary Collateral Function and Myocardial Ischemia. Circ. Cardiovasc. Interv..

[5] Faber J.E., Chilian W.M., Deindl E., van Royen N., Simons M. (2014). A brief etymology of the collateral circulation. Arter. Thromb. Vasc. Biol..

[6] Lasch M., Nekolla K., Klemm A.H., Buchheim J.-I., Pohl U., Dietzel S., Deindl E. (2019). Estimating hemodynamic shear stress in murine peripheral collateral arteries by two-photon line scanning. Mol. Cell. Biochem..

[7] Lasch M., Kleinert E.C., Meister S., Kumaraswami K., Buchheim J.-I., Grantzow T., Lautz T., Salpisti S., Fischer S., Troidl K. (2019). Extracellular RNA released due to shear stress controls natural bypass growth by mediating mechanotransduction in mice. Blood.

[8] Chillo O., Kleinert E.C., Lautz T., Lasch M., Pagel J.-I., Heun Y., Troidl K., Fischer S., Caballero-Martinez A., Mauer A. (2016). Perivascular Mast Cells Govern Shear Stress-Induced Arteriogenesis by Orchestrating Leukocyte Function. Cell Rep..

[9] Hoefer I.E., van Royen N., Rectenwald J.E., Deindl E., Hua J., Jost M., Grundmann S., Voskuil M., Ozaki C.K., Piek J.J. (2004). Arteriogenesis proceeds via ICAM-1/Mac-1- mediated mechanisms. Circ. Res..

[10] Deindl E., Ziegelhöffer T., Kanse S.M., Fernandez B., Neubauer E., Carmeliet P., Preissner K.T., Schaper W. (2003). Receptor-independent role of the urokinase-type plasminogen activator during arteriogenesis. FASEB J..

[11] Kluever A.K., Braumandl A., Fischer S., Preissner K.T., Deindl E. (2019). The Extraordinary Role of Extracellular RNA in Arteriogenesis, the Growth of Collateral Arteries. Int. J. Mol. Sci..

[12] Du Cheyne C., Tay H., De Spiegelaere W. (2020). The complex TIE between macrophages and angiogenesis. Anat. Histol. Embryol..

[13] Troidl C., Jung G., Troidl K., Hoffmann J., Mollmann H., Nef H., Schaper W., Hamm C.W., Schmitz-Rixen T. (2013). The temporal and spatial distribution of macrophage subpopulations during arteriogenesis. Curr. Vasc. Pharm..

[14] Holers V.M., Thurman J.M. (2004). The alternative pathway of complement in disease: Opportunities for therapeutic targeting. Mol. Immunol..

[15] Vogel C.W., Fritzinger D.C. (2007). Humanized cobra venom factor: Experimental therapeutics for targeted complement activation and complement depletion. Curr. Pharm. Des..

[16] Merle N.S., Church S.E., Fremeaux-Bacchi V., Roumenina L.T. (2015). Complement System Part I-Molecular Mechanisms of Activation and Regulation. Front. Immunol..

[17] Walport M.J. (2001). Complement. First of two parts. N. Engl. J. Med..

[18] Vogel C.W. (2020). The Role of Complement in Myocardial Infarction Reperfusion Injury: An Underappreciated Therapeutic Target. Front. Cell Dev. Biol..

[19] Huber-Lang M., Sarma J.V., Zetoune F.S., Rittirsch D., A Neff T., McGuire S.R., Lambris J., Warner R.L., A Flierl M., Hoesel L.M. (2006). Generation of C5a in the absence of C3: A new complement activation pathway. Nat. Med..

[20] Vogel C.W., Bredehorst R., Fritzinger D.C., Grunwald T., Ziegelmüller P., Kock M.A. (1996). Structure and function of cobra venom factor, the complement-activating protein in cobra venom. Adv. Exp. Med. Biol..

[21] Till G.O., Morganroth M.L., Kunkel R., Ward P.A. (1987). Activation of C5 by cobra venom factor is required in neutrophil-mediated lung injury in the rat. Am. J. Pathol..

[22] Morgan B.P., Harris C.L. (2003). Complement therapeutics; history and current progress. Mol. Immunol..

[23] Bossi F., Tripodo C., Rizzi L., Bulla R., Agostinis C., Guarnotta C., Munaut C., Baldassarre G., Papa G., Zorzet S. (2014). C1q as a unique player in angiogenesis with therapeutic implication in wound healing. Proc. Natl. Acad. Sci. USA.

[24] Langer H.F., Chung K.-J., Orlova V.V., Choi E.Y., Kaul S., Kruhlak M.J., Alatsatianos M., DeAngelis R.A., Roche P.A., Magotti P. (2010). Complement-mediated inhibition of neovascularization reveals a point of convergence between innate immunity and angiogenesis. Blood.

[25] Nozaki M., Raisler B.J., Sakurai E., Sarma J.V., Barnum S.R., Lambris J.D., Chen Y., Zhang K., Ambati B.K., Baffi J.Z. (2006). Drusen complement components C3a and C5a promote choroidal neovascularization. Proc. Natl. Acad. Sci. USA.

[26] Candinas D., Lesnikoski B.-A., Robson S.C., Miyatake T., Scesney S.M., Marsh H.C., Ryan U.S., Dalmasso A.P., Hancock W.W., Bach F.H. (1996). Effect of repetitive high-dose treatment with soluble complement receptor type 1 and cobra venom factor on discordant xenograft survival. Transplantation.

[27] Nording H., Baron L., Haberthür D., Emschermann F., Mezger M., Sauter M., Sauter R., Patzelt J., Knoepp K., Nording A. (2021). The C5a/C5a receptor 1 axis controls tissue neovascularization through CXCL4 release from platelets. Nat. Commun..

[28] Bongrazio M., Pries A.R., Zakrzewicz A. (2003). The endothelium as physiological source of properdin: Role of wall shear stress. Mol. Immunol..

[29] Chen J.Y., Cortes C., Ferreira V.P. (2018). Properdin: A multifaceted molecule involved in inflammation and diseases. Mol. Immunol..

[30] Fischetti F., Tedesco F. (2006). Cross-talk between the complement system and endothelial cells in physiologic conditions and in vascular diseases. Autoimmunity.

[31] Turner N.A., Moake J. (2013). Assembly and activation of alternative complement components on endothelial cell-anchored ultra-large von Willebrand factor links complement and hemostasis-thrombosis. PLoS ONE.

[32] Turner N., Nolasco L., Nolasco J., Sartain S., Moake J. (2014). Thrombotic microangiopathies and the linkage between von Willebrand factor and the alternative complement pathway. Semin. Thromb. Hemost..

[33] Wu F., Zou Q., Ding X., Shi D., Zhu X., Hu W., Liu L., Zhou H. (2016). Complement component C3a plays a critical role in endothelial activation and leukocyte recruitment into the brain. J. Neuroinflamm..

[34] Laudes I.J., Chu J.C., Huber-Lang M., Guo R.-F., Riedemann N.C., Sarma J.V., Mahdi F., Murphy H.S., Speyer C., Lu K.T. (2002). Expression and function of C5a receptor in mouse microvascular endothelial cells. J. Immunol..

[35] Scholz D., Ito W., Fleming I., Deindl E., Sauer A., Wiesnet M., Busse R., Schaper J. (2000). Ultrastructure and molecular histology of rabbit hind-limb collateral artery growth (arteriogenesis). Virchows Arch..

[36] Vogel C.W., Fritzinger D.C. (2010). Cobra venom factor: Structure, function, and humanization for therapeutic complement depletion. Toxicon.

[37] Vogel C.W., Müller-Eberhard H.J. (1984). Cobra venom factor: Improved method for purification and biochemical characterization. J. Immunol. Methods.

[38] Pickering R.J., Wolfson M.R., Good R.A., Gewurz H. (1969). Passive hemolysis by serum and cobra venom factor: A new mechanism inducing membrane damage by complement. Proc. Natl. Acad. Sci. USA.

[39] McCall C.E., De Chatelet L.R., Brown D., Lachmann P. (1974). New biological activity following intravascular activation of the complement cascade. Nature.

[40] Schmid E., Warner R.L., Crouch L.D., Friedl H.P., Till G.O., Hugli T.E., Ward P.A. (1997). Neutrophil chemotactic activity and C5a following systemic activation of complement in rats. Inflammation.

[41] Mitchell R.H., McClelland R.M., Kampschmidt R.F. (1982). Comparison of neutrophilia induced by leukocytic endogenous mediator and by cobra venom factor. Proc. Soc. Exp. Biol. Med..

[42] Xu G., Feng Y., Li D., Zhou Q., Chao W., Zou L. (2018). Importance of the Complement Alternative Pathway in Serum Chemotactic Activity During Sepsis. Shock.

[43] Yun S.H., Sim E.-H., Goh R.-Y., Park J.-I., Han J.-Y. (2016). Platelet Activation: The Mechanisms and Potential Biomarkers. Biomed. Res. Int..

[44] Bakogiannis C., Sachse M., Stamatelopoulos K., Stellos K. (2019). Platelet-derived chemokines in inflammation and atherosclerosis. Cytokine.

[45] Chatterjee M., Gawaz M. (2013). Platelet-derived CXCL12 (SDF-1α): Basic mechanisms and clinical implications. J. Thromb. Haemost..

[46] Yang D., Kastin A.J. (2013). Chapter 85-Anaphylatoxins. Handbook of Biologically Active Peptides.

[47] Ehrengruber M.U., Geiser T., Deranleau D.A. (1994). Activation of human neutrophils by C3a and C5A. Comparison of the effects on shape changes, chemotaxis, secretion, and respiratory burst. FEBS Lett..

[48] Gaudenzio N., Sibilano R., Marichal T., Starkl P., Reber L.L., Cenac N., McNeil B.D., Dong X., Hernandez J.D., Sagi-Eisenberg R. (2016). Different activation signals induce distinct mast cell degranulation strategies. J. Clin. Investig..

[49] Hartmann K., Henz B.M., Krüger-Krasagakes S., Köhl J., Burger R., Guhl S., Haase I., Lippert U., Zuberbier T. (1997). C3a and C5a stimulate chemotaxis of human mast cells. Blood.

[50] Reichel C.A., Uhl B., Lerchenberger M., Puhr-Westerheide D., Rehberg M., Liebl J., Khandoga A., Schmalix W., Zahler S., Deindl E. (2011). Urokinase-type plasminogen activator promotes paracellular transmigration of neutrophils via Mac-1, but independently of urokinase-type plasminogen activator receptor. Circulation.

[51] Pipp F., Heil M., Issbrücker K., Ziegelhoeffer T., Martin S., Van Den Heuvel J., Weich H., Fernandez B., Golomb G., Carmeliet P. (2003). VEGFR-1-selective VEGF homologue PlGF is arteriogenic: Evidence for a monocyte-mediated mechanism. Circ. Res..

[52] Ito W.D., Arras M., Winkler B., Scholz D., Schaper J., Schaper W. (1997). Monocyte chemotactic protein-1 increases collateral and peripheral conductance after femoral artery occlusion. Circ. Res..

[53] Wynn T.A., Vannella K.M. (2016). Macrophages in Tissue Repair, Regeneration, and Fibrosis. Immunity.

[54] Arras M., Ito W.D., Scholz D., Winkler B., Schaper J., Schaper W. (1998). Monocyte activation in angiogenesis and collateral growth in the rabbit hindlimb. J. Clin. Investig..

[55] Limbourg A., Korff T., Napp L.C., Schaper W., Drexler H., Limbourg F. (2009). Evaluation of postnatal arteriogenesis and angiogenesis in a mouse model of hind-limb ischemia. Nat. Protoc..

[56] Ing M., Hew B.E., Fritzinger D.C., Delignat S., Lacroix-Desmazes S., Vogel C.-W., Rayes J. (2018). Absence of a neutralizing antibody response to humanized cobra venom factor in mice. Mol. Immunol..

[57] Vogel C.W., Gorsuch B., Stahl G., Vogel C.-W. (2015). Complement depletion with humanised cobra venom factor: Efficacy in preclinical models of vascular diseases. Thromb. Haemost..

[58] Vogel C.W., Finnegan P.W., Fritzinger D.C. (2014). Humanized cobra venom factor: Structure, activity, and therapeutic efficacy in preclinical disease models. Mol. Immunol..

