# Supplementary material for: Cobra Venom Factor Boosts Arteriogenesis in Mice
Source: Int J Mol Sci. 2022 Jul 30;23(15):8454. doi: 10.3390/ijms23158454 (PMC9368946; doi:10.3390/ijms23158454)
Supplement: Supplementary file 1 [file ijms-23-08454-s001.zip › ijms-1806812-supplementary.pdf]

International Journal of *Molecular Sciences*

MDPI

Article

Cobra Venom Factor Boosts Arteriogenesis in Mice

Philipp Götz[1,2,†], Sharon O. Azubuike-Osu[1,2,3,†], Anna Braumandl[1,2], Christoph Arnholdt[1,2], Matthias Kübler[1,2], Lisa Richter[4], Manuel Lasch[1,2,5], Lisa Bobrowski[1,2], Klaus T. Preissner[6], and Elisabeth Deindl[1,2,*]

1 Walter-Brendel-Centre of Experimental Medicine, University Hospital, Ludwig-Maximilians-Universität München, 81377, Munich, Germany; Sharon.Azubuike-Osu@med.uni-muenchen.de (S.O.A.-O.); P.Goetz@med.uni-muenchen.de (P.G.); Anna.Braumandl@med.uni-muenchen.de (A.B.); christoph.arnholdt@med.uni-muenchen.de (C.A.); Matthias.Kuebler@med.uni-muenchen.de (M.K.); manuel_lasch@gmx.de (M.L.); Lisa.Bobrowski@med.uni-muenchen.de (L.B.); Elisabeth.Deindl@med.uni-muenchen.de (E.D.)
2 Biomedical Center, Institute of Cardiovascular Physiology and Pathophysiology, Ludwig-Maximilians-Universität München, 82152, Planegg-Martinsried, Germany
3 Department of Physiology, Faculty of Basic Medical Sciences, College of Medicine, Alex Ekwueme Federal University Ndufu Alike, Ebonyi, Nigeria; sharon.eboagwu@funai.edu.ng
4 Flow Cytometry Core Facility, Biomedical Center, Ludwig-Maximilians-Universität München, 82152, Planegg-Martinsried, Germany; l.richter@med.uni-muenchen.de
5 Department of Otorhinolaryngology, Head and Neck Surgery, University Hospital, Ludwig-Maximilians-Universität München, 81377 Munich, Germany
6 Department of Cardiology, Kerckhoff-Heart Research Institute, Faculty of Medicine, Justus Liebig University, 35392, Giessen, Germany; Klaus.T.Preissner@biochemie.med.uni-giessen.de
* Correspondence: Elisabeth.Deindl@med.uni-muenchen.de; Tel.: +49 (0) 89 2180 76504
† These authors contributed equally to this work.

Citation: Götz, P.; Azubuike-Osu, S. O.; Braumandl, A.; Arnholdt, C.; Kübler, M.; Richter, L.; Lasch, M.; Bobrowski, L.; Preissner, K. T.; Deindl, E. Cobra Venom Factor Boosts Arteriogenesis in Mice. *Int. J. Mol. Sci.* **2022**, *23*, 8454. https://doi.org/10.3390/ijms23158454

Academic Editor: Steve Peigneur

Received: 23 June 2022
Accepted: 27 July 2022
Published: 30 July 2022

Publisher's Note: MDPI stays neutral with regard to jurisdictional claims in published maps and institutional affiliations.

Copyright: © 2022 by the authors. Licensee MDPI, Basel, Switzerland. This article is an open access article distributed under the terms and conditions of the Creative Commons Attribution (CC BY) license (https://creativecommons.org/licenses/by/4.0/).

Supplementary Material

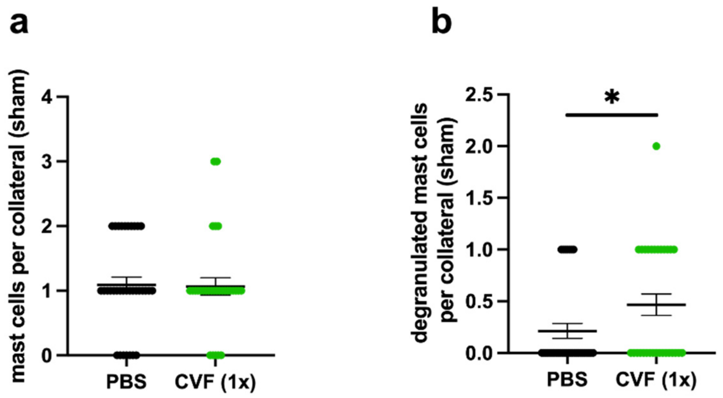

Figure S1. Single-dose application of cobra venom factor (CVF (1x)) does not influence the number but the degranulation of perivascular mast cells of resting collateral arteries. The scatter plots represent (**a**) the number of perivascular mast cells per collateral in adductor muscles 8h after femoral artery sham operation (sham) of phosphate-buffered saline (PBS)- and CVF (1x)-treated mice, as well as (**b**) the number of degranulated mast cells per collateral. Data are means ± S.E.M., n = 3 mice per group, n > 10 values per group, * $p < 0.05$ (PBS vs CVF (1x)), by unpaired student's *t*-test.

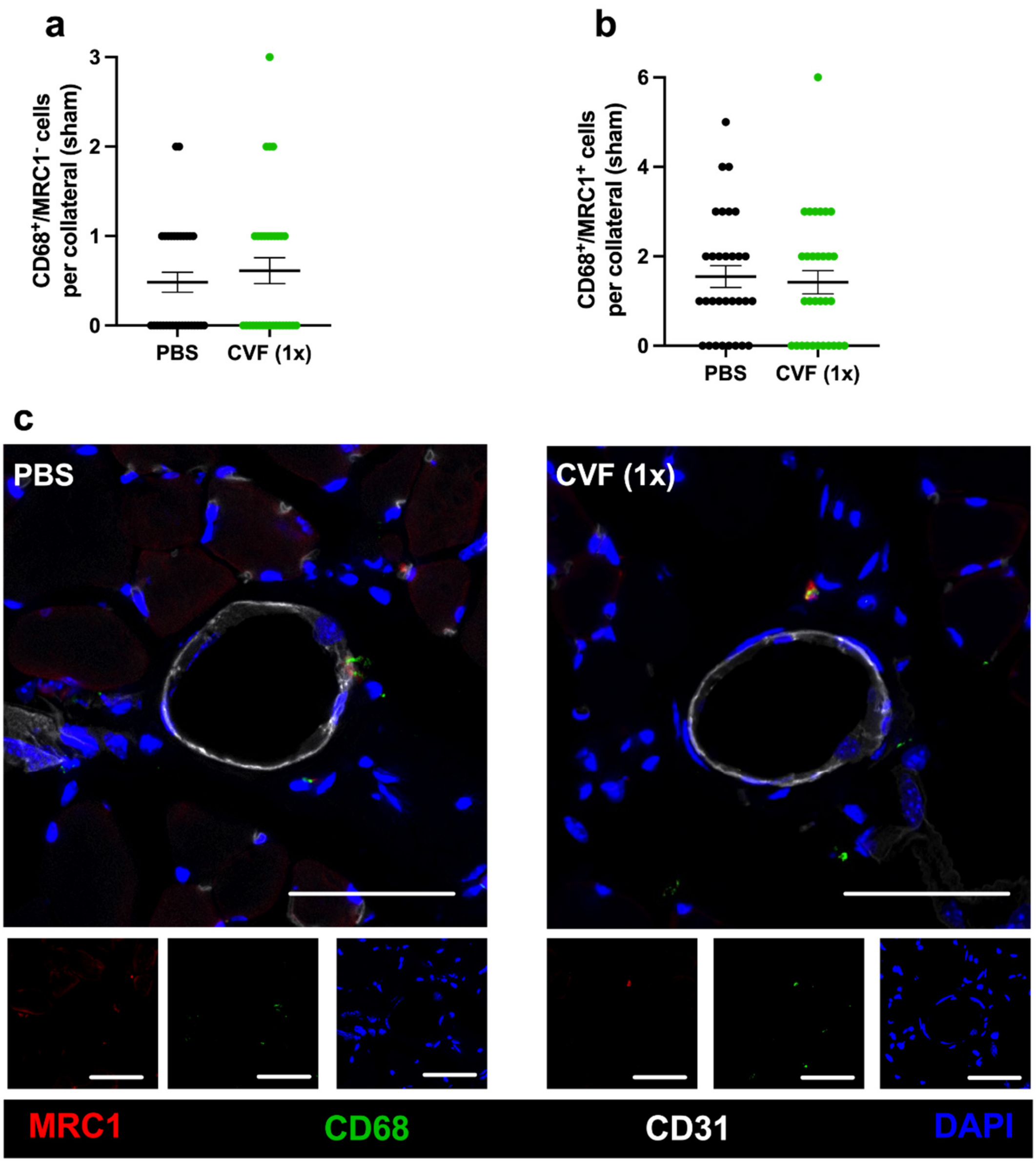

Figure S2. Single-dose cobra venom factor (CVF (1x)) application has no impact on the number of macrophages and their polarization upon sham operation. The scatter plots display the number of (**a**) $CD68^+/MRC1^-$ (mannose receptor C-type 1) and (**b**) $CD68^+/MRC1^+$ cells per resting collateral in adductor muscles of phosphate-buffered saline (PBS)- and CVF (1x)-treated mice collected 7 days after sham operation. Data are means ± S.E.M., n = 5 mice per group, n > 10 values per group, $p \geq 0.05$ (PBS vs CVF (1x)), by unpaired student's *t*-test. (**c**) Representative immunofluorescence images of adductor muscles of PBS- (left images) and CVF (1x)-treated (right images) mice collected 7 days after sham operation. Images of single and merged channels showing macrophages labeled with antibodies against CD68 (green) and MRC1 (red) in the perivascular space of collaterals. The endothelial cell marker CD31 was used to depict collaterals, and DAPI (blue) to label nucleic DNA. Scale bars: 50 μm.
